# A novel artificial intelligence network to assess the prognosis of gastrointestinal cancer to immunotherapy based on genetic mutation features

**DOI:** 10.3389/fimmu.2024.1428529

**Published:** 2024-06-27

**Authors:** Bicheng Ye, Zhongyan Li, Qiqi Wang

**Affiliations:** ^1^ School of Clinical Medicine, Yangzhou Polytechnic College, Yangzhou, China; ^2^ Department of Geriatric Medicine, Huai’an Hospital Affiliated to Yangzhou University (The Fifth People’s Hospital of Huai’an), Huai’an, China; ^3^ Department of Gastroenterology, Wenzhou Central Hospital, Wenzhou, China; ^4^ Department of Gastroenterology, The Dingli Clinical College of Wenzhou Medical University, Wenzhou, China; ^5^ Department of Gastroenterology, The Second Afliated Hospital of Shanghai University, Wenzhou, China

**Keywords:** artificial intelligence, gastrointestinal cancer, genomic mutation, immunotherapy, immune landscape

## Abstract

**Background:**

Immune checkpoint inhibitors (ICIs) have revolutionized gastrointestinal cancer treatment, yet the absence of reliable biomarkers hampers precise patient response prediction.

**Methods:**

We developed and validated a genomic mutation signature (GMS) employing a novel artificial intelligence network to forecast the prognosis of gastrointestinal cancer patients undergoing ICIs therapy. Subsequently, we explored the underlying immune landscapes across different subtypes using multiomics data. Finally, UMI-77 was pinpointed through the analysis of drug sensitization data from the Genomics of Drug Sensitivity in Cancer (GDSC) database. The sensitivity of UMI-77 to the AGS and MKN45 cell lines was evaluated using the cell counting kit-8 (CCK8) assay and the plate clone formation assay.

**Results:**

Using the artificial intelligence network, we developed the GMS that independently predicts the prognosis of gastrointestinal cancer patients. The GMS demonstrated consistent performance across three public cohorts and exhibited high sensitivity and specificity for 6, 12, and 24-month overall survival (OS) in receiver operating characteristic (ROC) curve analysis. It outperformed conventional clinical and molecular features. Low-risk samples showed a higher presence of cytolytic immune cells and enhanced immunogenic potential compared to high-risk samples. Additionally, we identified the small molecule compound UMI-77. The half-maximal inhibitory concentration (IC50) of UMI-77 was inversely related to the GMS. Notably, the AGS cell line, classified as high-risk, displayed greater sensitivity to UMI-77, whereas the MKN45 cell line, classified as low-risk, showed less sensitivity.

**Conclusion:**

The GMS developed here can reliably predict survival benefit for gastrointestinal cancer patients on ICIs therapy.

## Introduction

Gastrointestinal cancers constitute a significant health challenge worldwide, accounting for 26% of all cancer diagnoses and 35% of cancer-related fatalities (1). Immune checkpoint inhibitors (ICIs) have emerged as a potentially effective therapeutic strategy for a variety of cancer types, including those of the gastrointestinal cancer (2, 3). However, the response rate to ICIs is limited, varying from 10–20% across different tumor types (3, 4). Consequently, the development of biomarkers capable of accurately identifying patients who are likely to benefit from ICIs therapy is of paramount importance.

Microsatellite instability (MSI), a genetic indicator of tumor responsiveness to ICIs, stands as the sole validated biomarker in clinical trials for gastrointestinal cancers (5, 6). However, MSI-high tumors are relatively uncommon, representing only 0–5% of all metastatic gastrointestinal cancer cases (7). Programmed death ligand-1 (PD-L1) expression is another commonly assessed biomarker for the application of ICIs, but its predictive value is inconsistent across different trials due to heterogeneity and variability of expression and detection (8, 9). Another promising biomarker under investigation is the tumor mutation burden (TMB), which has demonstrated a correlation with response to ICIs in recent research (10). However, TMB is not a reliable biomarker for gastrointestinal cancer (11). Not all mutations have the same immunogenic impact, and some mutations, such as CDKN2A, ARID1A, ARID1B, ARID2, ERBB4, and ZFHX3, may modulate the outcomes of ICIs treatment in positive or negative ways (12–15). Besides, In the context of gastrointestinal tumors, certain genetic mutations are closely linked to the effectiveness of immunotherapy. Mutations in the AKT1 and CDH1 genes have been associated with primary resistance to ICIs (16). These insights highlight the importance of gene mutations in predicting responses to immunotherapy and tailoring personalized treatment approaches for patients with gastrointestinal cancers. TMB scoring systems do not account for the differential effects of these mutations, limiting their predictive value for ICIs (17). To overcome this limitation, some studies have suggested refining the TMB algorithm (18) or constructing gene mutation-based signatures to improve the survival prediction of ICIs in gastrointestinal cancer (17, 19, 20).

Machine learning and deep learning are powerful tools for solving complex problems in medicine using large clinical data sets (20). These methods have demonstrated their achievements and efficiency in prediction and clustering tasks (21). By applying these novel technologies, we can explore the mechanisms of therapy resistance at different levels, such as transcriptional, epigenetic, and translational levels, and find more clues to improve the efficacy of ICIs (22–24). Thus, we developed a novel artificial intelligence network that integrated traditional regression algorithms, machine learning, and deep learning, comprising a total of 22 algorithms and 297 algorithm combinations, greatly surpassing the previous 101 algorithm combinations (25). This comprehensive approach allows us to more accurately analyze and predict the outcomes of immunotherapy for gastrointestinal tumors.

In this work, we used genomic mutation information to develop and validate an artificial intelligence network-based genomic mutation signature (GMS). This study may provide guidance for immunotherapy treatment decisions and improve the clinical outcomes of gastrointestinal cancer.

## Methods

### Designing research studies and collecting data

**Figure 1:**
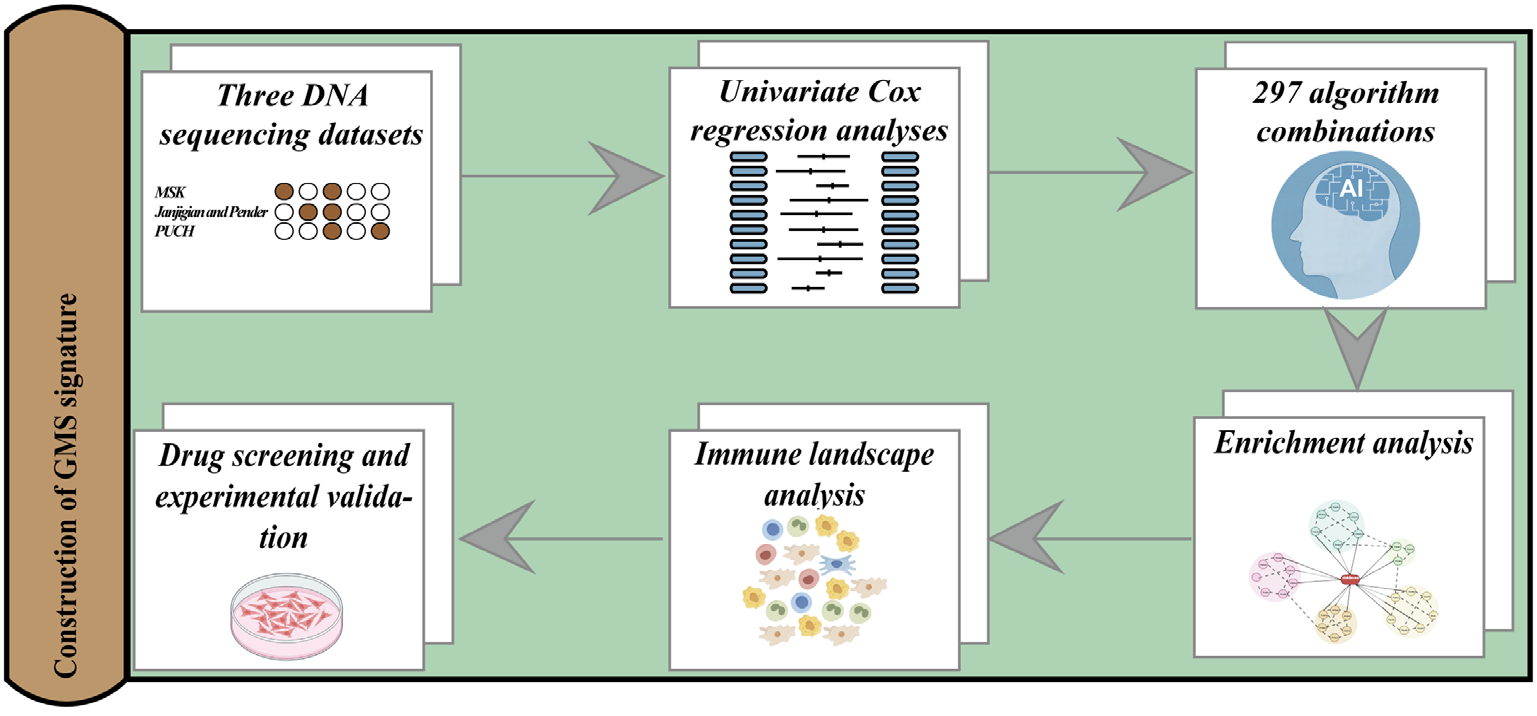
An illustration of the general workflow adopted in this study.

We present the overall study design in **Figure 1**. We collected 233 gastrointestinal cancer cases treated with ICIs from Memorial Sloan Kettering Cancer Center (MSK) as a training cohort to screen for mutations with prognostic potential and to construct a prognostic signature (11). We also obtained two independent validation cohorts of gastrointestinal cancers with ICIs treatment from public databases. The combined Janjigian and Pender cohort comprised 39 cases of metastatic chemotherapy-refractory esophagogastric cancer (26) and 9 cases of metastatic or advanced gastrointestinal cancer (27). The PUCH cohort consisted of 91 patients with gastrointestinal cancer (17). The patient enrollment criteria are as follows: (1) primary gastrointestinal cancers; (2) availability of gene mutation profiles and clinical annotations, including follow-up data; (3) receipt of at least one cycle of a CTLA-4 inhibitor, PD-1/PD-L1 inhibitor or combined treatment. Furthermore, we obtained somatic mutation data, mRNA expression profiles, and copy number variations (CNV) for a non-immunotherapy gastrointestinal cancer cohort consisting of 184 cases of esophageal cancer, 439 cases of gastric cancer, and 380 cases of colorectal cancer from The Cancer Genome Atlas (TCGA) database. The genomic and clinical data for the MSK cohort, the Janjigian and Pender cohorts, and the PUCH cohort, are openly available and were downloaded from the following sources: MSK cohort (http://www.cbioportal.org/study?id=tmb_mskcc_2018), Janjigian cohort (https://www.cbioportal.org/study/summary?id=egc_msk_2017), Pender cohort (http://clincancerres.aacrjournals.org/content/27/1/202.article-info), and PUCH cohort (https://www.bcgsc.ca/downloads/immunoPOG/). The data from the TCGA dataset are available for download at https://portal.gdc.cancer.gov/.

### Analysis of mutation data and evaluation of clinical outcomes

Tumor tissues from the MSK and Janjigian cohorts were subjected to sequencing using the MSK-IMPACT sequencing technique, which involved either a 341-gene panel, a 410-gene panel, or a 468-gene panel. For the Pender cohort, whole-genome sequencing (WGS) was utilized for tumor tissue analysis, and whole-exome sequencing (WES) was employed for the PUCH cohort. The mutated gene status was assigned a value of 1, and the wild-type gene status was assigned a value of 0. The primary survival endpoint considered was overall survival (OS). The clinical response was assessed per the Response Evaluation Criteria in Solid Tumors version 1.1. Durable clinical benefit (DCB) met the criteria for complete response (CR), partial response (PR), or stable disease (SD) persisting for ≥6 months. Conversely, no durable benefit (NDB) was defined as progressive disease (PD) criteria or SD <6 months (28).

### Artificial intelligence network-based signature generation

We constructed a novel artificial intelligence network based on 297 algorithm combinations, integrating 22 algorithms from traditional regression, machine learning, and deep learning. These algorithms included random survival forest (RSF), supervised principal components (SuperPC), oblique random survival forests (obliqueRSF), gradient boosting with component-wise linear models (GLMBoost), gradient boosting with regression trees (BlackBoost), stepwise Cox, recursive partitioning and regression trees (Rpart), parametric survival model (Survreg), Ranger, conditional inference trees (Ctree), least absolute shrinkage and selection operator (LASSO), partial least squares regression for Cox (plsRcox), survival support vector machine (survival-SVM), Ridge, elastic network (Enet), deephit survival neural network (DeepHit), deepsurv survival neural network (DeepSurv), cox-time survival neural network (CoxTime), extreme gradient boosting (XGBoost), Coxboost, CForest, and variable selection oriented LASSO bagging algorithm (VSOLassoBag). We developed the signature as follows: (1) Prognostic genes were identified via univariate Cox regression in the MSK cohort. (2) Initial signature discovery utilized an artificial intelligence network in the MSK cohort. (3) Further testing of the network occurred in two validation cohorts (Janjigian/Pender and PUCH). (4) Harrell’s concordance index (C-index) evaluated each model’s performance across all cohorts. The model with the maximal average C-index across the test cohorts was deemed optimal based on its superior predictive ability. The source code and specific parameters of this artificial intelligence network can be found at the following GitHub repository: https://github.com/miaolab1998/AI_network/tree/main.

### Functional annotation of the GMS

We collected immune modulators from a previous study (29). We used four algorithms to quantify immune infiltrating cells: the quanTIseq algorithm (30) of 11 immune cells, the estimating the proportions of immune and cancer cells (EPIC) algorithm (31) of eight immune cells, the Microenvironment Cell Populations-counter (MCPcounter) algorithm (32)of ten immune cells, and the Estimation of STromal and Immune cells in Malignant Tumours using Expression data (ESTIMATE) algorithm (33). We also acquired 29 classical immune signatures from the work of He et al. (34). The cytolytic activity scores (CYTs) were estimated using the geometric mean of GZMA and PRF1 (35). Employing the GSVA R package, which is grounded in the single-sample gene set enrichment analysis (ssGSEA) technique, we quantified the enrichment levels of the 29 immune signatures across each sample (36). Utilizing the GSVA method (36) and clusterprofiler (37) R packages, we executed gene set variation analysis (GSVA) and gene set enrichment analysis (GSEA) on the MSigDB database. We also used Metascape for enrichment analysis (38).

### Calculation of immunogenomic indicators

We obtained immunogenomic indicators from the pan-cancer immune landscape study (29). In summary, they established the intertumoral heterogeneity (ITH) score to quantify the subclonal genomic fraction, reflecting tumor genome segments unaccounted for by the dominant clone. This was determined via ABSOLUTE, a tool modeling tumor alterations including subclonal and clonal components with varying ploidies. CNV burden metrics were n_segs, indicating segment count per sample, and frac_altered, denoting proportion of bases diverging from baseline ploidy. The aneuploidy score aggregated altered chromosomal arms. Additionally, T-cell receptor (TCR) and B-cell receptor (BCR) diversity indices like Shannon entropy and richness were calculated from cancer RNA-seq data.

### Oncogenic pathway enrichment scores

From the study by Sanchez-Vega et al (39), we obtained ten canonical oncogenic pathways that include 187 oncogenes. We applied the GSVA method, facilitated by the GSVA R package (36), to calculate the enrichment scores for these pathways in each sample.

### Uncovering genomic mutational signatures

Employing the maftools R package, we conducted nonnegative matrix factorization (NMF) on a dataset of 96 trinucleotide context mutations from gastrointestinal cancer specimens, which were obtained from the TCGA. We then compared the resulting mutational landscape to the Catalogue of Somatic Mutations in Cancer (COSMIC), employing cosine similarity for the assessment.

### Drug prediction

We retrieved data on tumor cell line sensitivity to potential drugs and mutations from the Genomics of Drug Sensitivity in Cancer (GDSC) database. The cell line sensitivity was assessed using the lower half maximal inhibitory concentration (IC50) values of the respective drugs.

### Cell line culture

The human gastric cancer cell lines AGS and MKN45 were acquired from the Shanghai Institutes for Biological Sciences, part of the Chinese Academy of Sciences. MKN45 cells were grown in RPMI 1640 medium supplemented with 10% FBS and 1% penicillin-streptomycin. AGS cells were cultivated in Ham’s F-12 medium with the same supplements. The cells were incubated at 37°C with 5% CO2.

### CCK-8 detection

Cells were seeded into a 96-well plate at an optimal density of 5,000 cells per well. We treated the cells with different concentrations of UMI-77 and incubated them for 48 h and 72 h. We measured and recorded the absorbance value on the cell growth curve and calculated the IC50.

### Colony formation assay

1000 untreated cells were cultured in each well of a six-well plate, either with UMI-77, DMSO, or without any treatment, for a period of 2 weeks. Following this, colony formation was analyzed.

### Statistical analysis

Categorical data were examined with the chi-square test, and numerical data with the Wilcoxon test. Pearson test was employed for association analysis. Survival curves were generated with the Survival and survminer packages in R. Univariate and multivariate Cox regression analyses were performed to assess the GMS’s clinical factor independence. Receiver operator characteristic curve (ROC) and area under the ROC curve (AUC) were used to determine the predictive sensitivity and specificity for survival or response. Statistical significance was defined as a P value below 0.05, unless stated otherwise. All analyses were conducted using R version 4.2.3.

## Results

### Construction and valiation of the GMS

**Figure 2:**
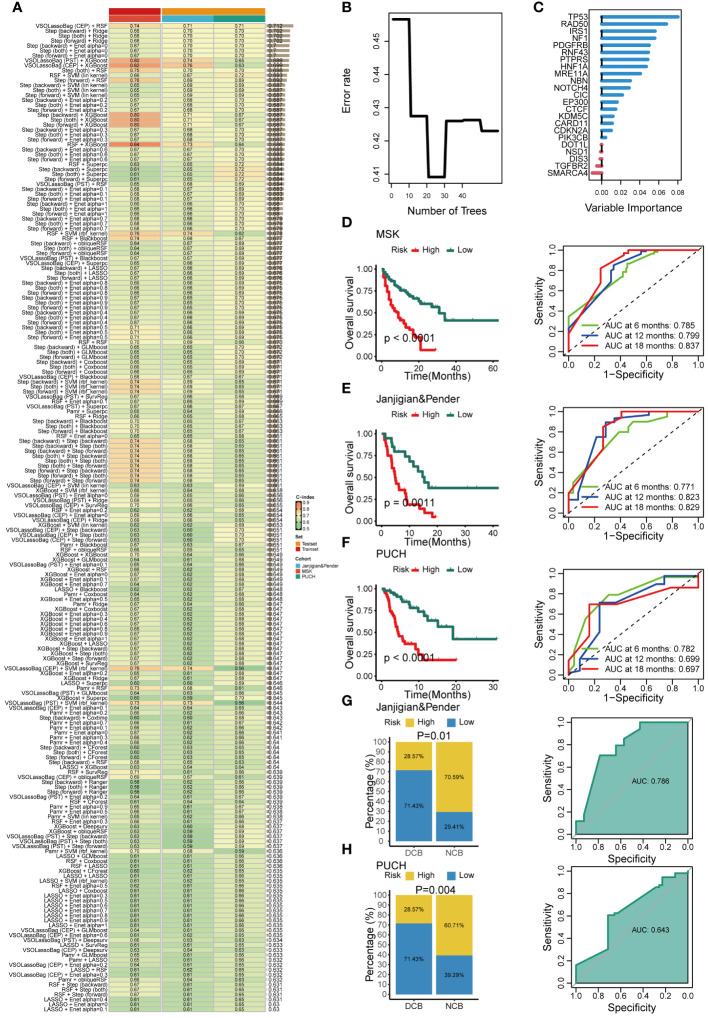
Development and validation of an artificial intelligence network using 297 algorithm combinations. **(A)** Evaluation and C-index computation for 297 prediction models across all validation datasets. **(B)** Determination of the number of trees by minimizing error. **(C)** Variable importance of the top 23 genes determined using the random survival forest (RSF) algorithm. **(D-F)** Kaplan-Meier survival analysis (left) and receiver operating characteristic (ROC) (right) curves for overall survival (OS) in the MSK **(D)**, Janjigian and Pender **(E)**, and PUCH **(F)** cohorts. **(G, H)** The correlation between genomic mutation signature (GMS) and response (left), as well as the ROC of GMS predicting clinical response (right) in the Janjigian and Pender cohort **(G)**, and PUCH cohort **(H)**.

The characteristics of patients in these three immunotherapeutic cohorts are detailed in **Supplementary Table 1**. The training cohort consisted of 233 gastrointestinal cancer patients (esophagogastric cancer, N = 123; colorectal cancer, N = 110) from MSK who received ICIs. We identified 74 prognostic genes through univariate Cox analysis and selected seed genes with a mutation frequency greater than 3%. These genes were then subjected to our artificial intelligence network to construct a GMS. The optimal model, comprising a combination of VSOLassoBag and RSF, was determined based on its highest average C-index (C-index = 0.71) among the 297 algorithm combinations evaluated through 10-fold cross-validation (**Figure 2A**). The VSOLassoBag algorithm selected 23 genes based on curve elbow point detection (CEP) method and used them to construct the most reliable GMS by RSF (**Figures 2B, C**). The GMS score was determined for each participant and stratified them into high and low-risk groups per the training set (median GMS score = 16.65). The high-risk group had markedly inferior OS versus low-risk (all p < 0.05) across all cohorts (**Figures 2D–F**). In the MSK cohort, 6-month AUC = 0.785, 12-month AUC = 0.799, and 18-month AUC = 0.837 (**Figure 2D**). In the Janjigian&Pender cohort, 6-month AUC = 0.771, 12-month AUC = 0.823, and 18-month AUC = 0.829 (**Figure 2E**). In the PUCH cohort, 6-month AUC = 0.782, 12-month AUC = 0.699, and 18-month AUC = 0.697 (**Figure 2F**). The time-dependent ROC curves demonstrated the strong and consistent performance of the GMS across all cohorts. In the two test cohorts, a notable number of patients with DCB had low GMS scores (all p < 0.05). The ROC analyses in these cohorts suggested that the GMS could be a valuable predictive biomarker for immunotherapy clinical benefit, with AUCs of 0.786 and 0.643, respectively (**Figures 2G, H**). These findings suggest the GMS may act as a robust predictor of responses and outcomes for gastrointestinal cancer patients undergoing immunotherapy.

### The strong predictive performance of GMS

**Figure 3:**
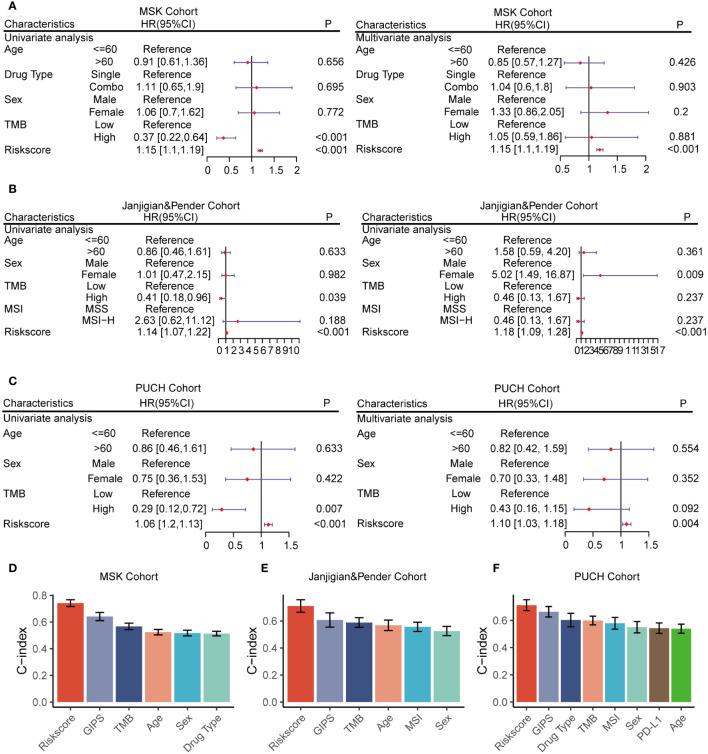
Univariate and multivariate Cox regression analyses of the GMS and other characteristics. **(A)** GMS subjected to univariate and multivariate Cox regression analyses in the MSK cohort. **(B)** GMS subjected to univariate and multivariate Cox regression analyses in the Janjigian and Pender cohort. **(C)** GMS subjected to univariate and multivariate Cox regression analyses in the PUCH cohort. **(D)** Comparison of GMS performance with other clinical and molecular variables for prognosis prediction in the MSK cohort. **(E)** Comparison of GMS performance with other clinical and molecular variables for prognosis prediction in the Janjigian and Pender cohort. **(F)** Comparison of GMS performance with other clinical and molecular variables for prognosis prediction in the PUCH cohort.

Univariate and multivariate Cox regression analyses were conducted across all cohorts to evaluate GMS as an independent predictor of OS in immunotherapy patients. In the univariate and multivariate analyses, GMS emerged as a robust predictor, not affected by adjustments for age, gender, drug category, MSI, PDL-1, and TMB (**Figures 3A–C**), solidifying its predictive utility in prognosis. To compare the predictive superiority of GMS, we assessed it against common clinical traits and molecular features. GMS exhibited significantly higher accuracy compared to other variables, such as age, gender, drug type, the genomic mutation signature of immunotherapy for gastrointestinal tumors identified in previous studies (GIPS) (17), TMB, MSI, and PD-L1, across all three cohorts (**Figures 3D–F**). These results indicate that our GMS holds promise as a reliable surrogate for predicting the prognosis of gastrointestinal cancer patients receiving immunotherapy in clinical practice.

### Potential biological peculiarities of the GMS

**Figure 4:**
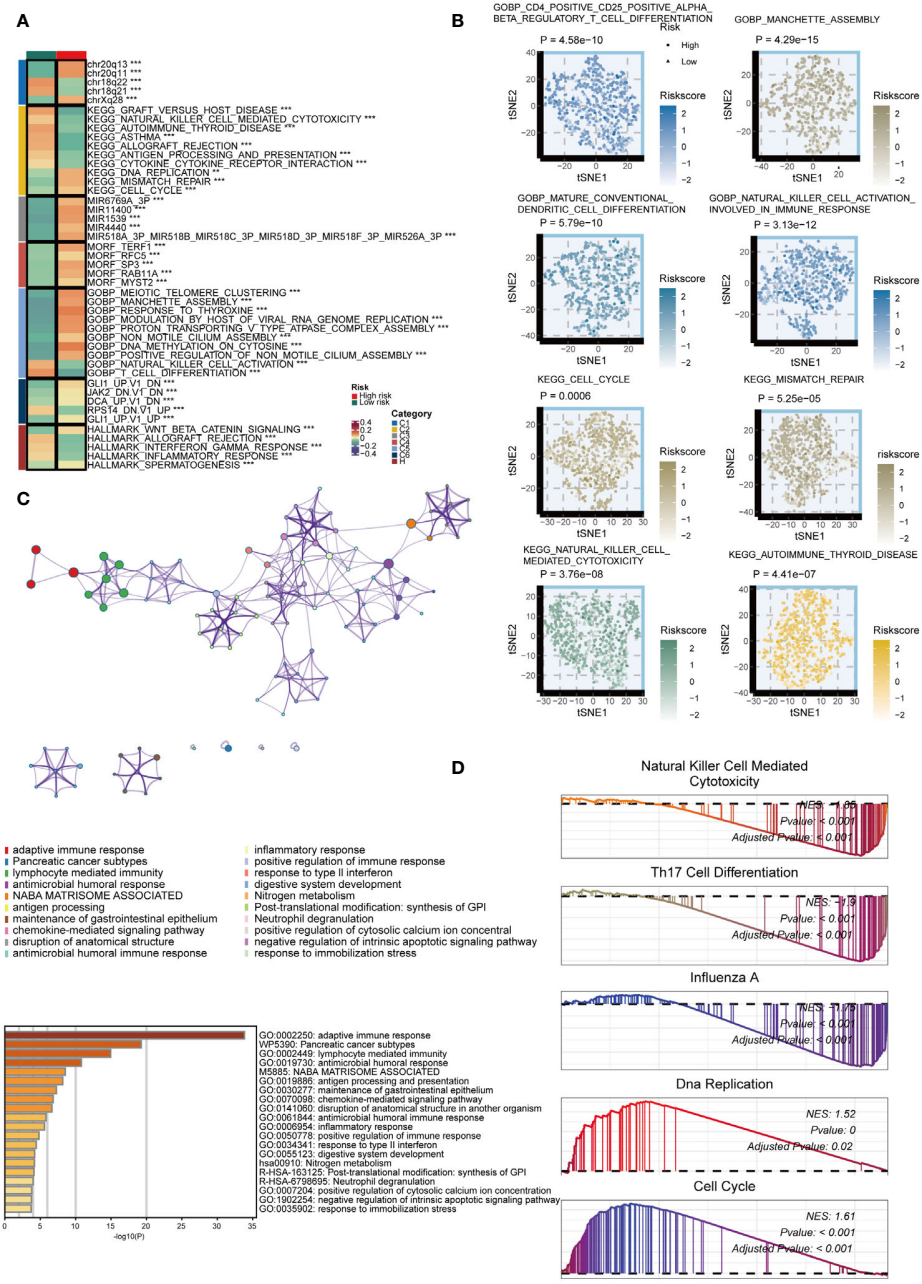
Biological peculiarities of the GMS in the TCGA dataset. **(A)** Outlining the biological characteristics of two groups based on GMS using MsigDB-based Gene Set Variation Analysis (GSVA) in the TCGA dataset. **(B)** T-distributed Stochastic Neighbor Embedding (t-SNE) plot to illustrate differences in pathway activity between two GMS groups based on Gene Ontology (GO) and Kyoto Encyclopedia of Genes and Genomes (KEGG) terms. **(C)** Metascape-based enrichment analysis of high expression genes in the low-risk group. **(D)** Gene Set Enrichment Analysis (GSEA) for GO and KEGG terms to investigate biological pathways associated with GMS in the TCGA dataset. **p < 0.01; ***p < 0.001.

We examined the biological mechanisms of GMS in the TCGA dataset. We noted that the GMS displayed a negative correlation with numerous immune pathways, including graft-versus-host disease, natural killer cell-mediated cytotoxicity, cytokine-cytokine receptor interaction, antigen processing, asthma, allograft rejection, and autoimmune thyroid disease pathways (**Figure 4A**). Conversely, the GMS showed a positive correlation with several tumorigenic pathways, such as DNA replication, mismatch repair, manchette assembly, cytosine DNA methylation, meiotic telomere clustering, and cell cycle pathways (**Figure 4A**). Further analysis revealed significant differences in immunological and tumorigenic pathways between the high- and low-risk groups (**Figure 4B**). The genes with high expression in the low-risk group were enriched in immune activation and infiltration pathways (**Figure 4C**). GSEA using Kyoto Encyclopedia of Genes and Genomes (KEGG) terms showed the low-risk group had enrichment in NK cell cytotoxicity, Th17 cell differentiation, and influenza A, as anticipated (**Figure 4D**). In contrast, the high-risk group displayed enrichment in DNA replication and cell cycle pathways. These results indicate that a lower GMS score tends to be associated with an inflammatory environment.

### Extrinsic immune landscapes of the GMS

**Figure 5:**
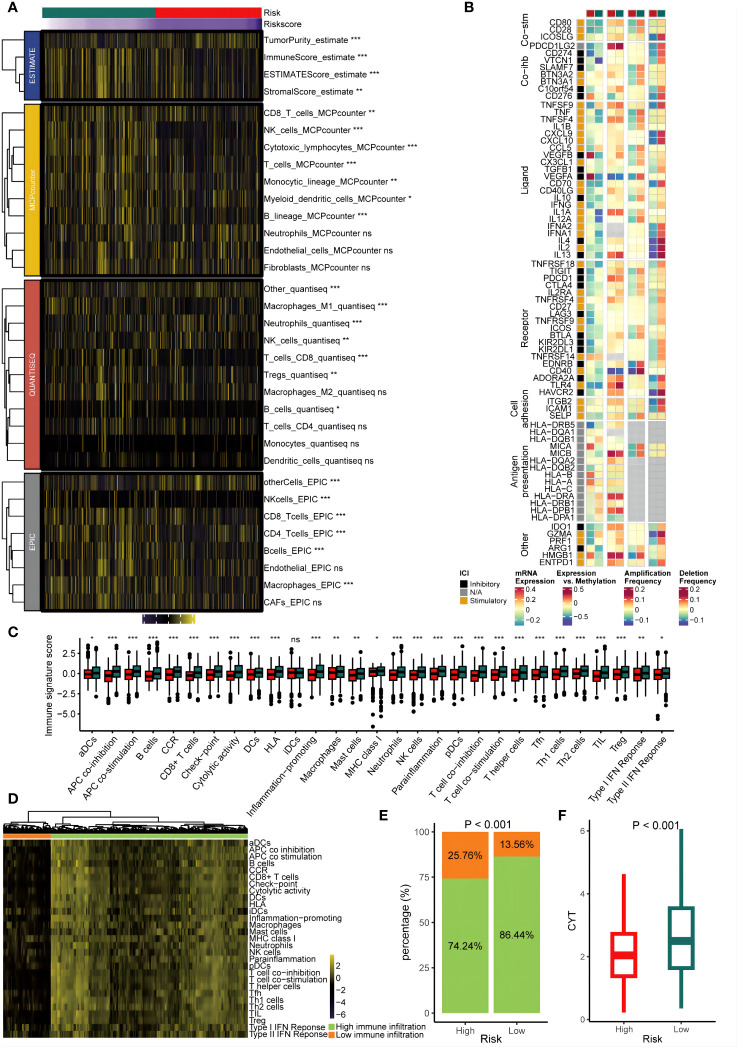
Immune infiltrating characteristics of the GMS in the cohort from TCGA. **(A)** The relationship between the GMS and infiltrating immune cell populations. **(B)** The association between the GMS and immune modulatory factors. **(C)** The relationship between the GMS and 29 immune signatures score. **(D)** Unsupervised clustering based on 29 immune signatures. **(E)** The proportions of high and low immune infiltration were estimated in both the high-risk and low-risk groups. **(F)** A comparison of the cytolytic activity scores (CYTs) score was conducted betweenthe high-risk and low-risk groups. NS, no significant; *p < 0.05; **p < 0.01; ***p < 0.001.

We assessed the GMS as an indicator of immune status by analyzing its association with infiltration of immune cells and expression of immune checkpoints. **Figures 5A, B** show that the low-risk group had increased infiltration of immune cells and immune modulatory activity in the TCGA dataset. Comparison of the 29 immune signatures between groups revealed that the low-risk group had higher prevalence of immune cells including CD8+ T cells (p < 0.05) (**Figure 5C**). To determine if the risk groups corresponded to low and high infiltration cohorts, unsupervised clustering of the 29 immune signatures for TCGA patients was performed. This identified two distinct immune patterns: high and low immune infiltration (**Figure 5D**). Notably, the low-risk group was more common in the high infiltration cluster (p < 0.05) (**Figure 5E**). Furthermore, low-risk tumors were linked to significantly higher CYT scores (p < 0.05) (**Figure 5F**). These results implied a relatively inflamed and immunostimulatory microenvironment, which may be amenable to immunotherapy (40).

### Intrinsic immune landscapes of the GMS

**Figure 6:**
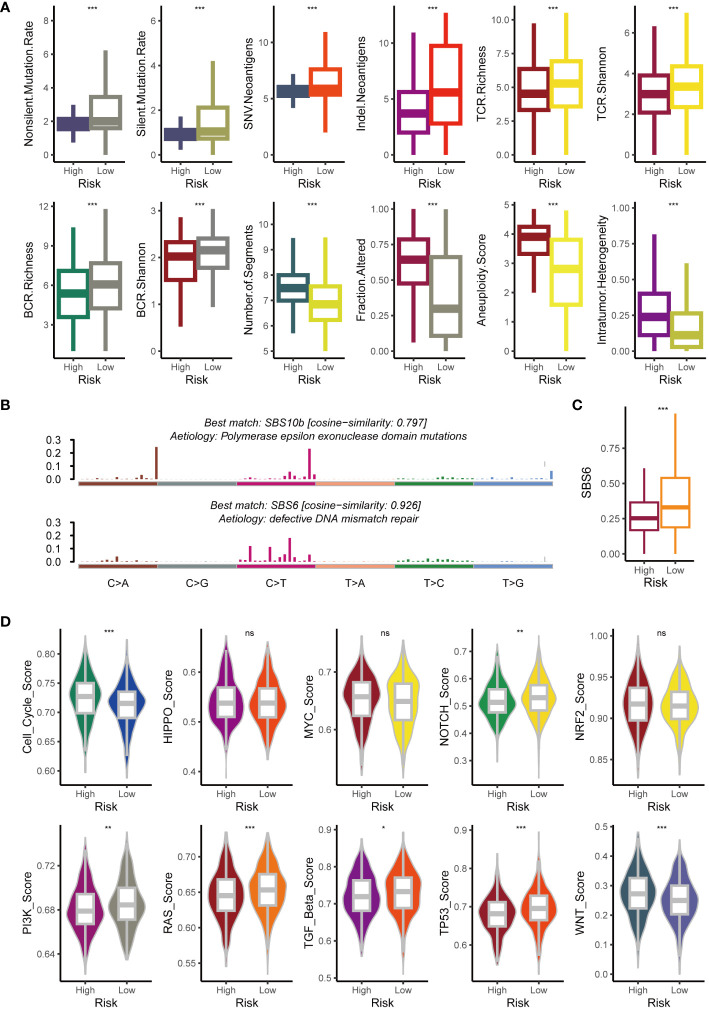
Exploration of potential intrinsic immune response and escape landscapes in the high-risk and low-risk groups. **(A)** Comparison of immunogenomic markers between the high-risk and low-risk groups. **(B)** Analysis of mutational activities of two extracted mutational signatures. **(C)** Comparison of the SBS6 signature activity between high-risk and low-risk groups. **(D)** Comparison of enrichment scores for 10 oncogenic pathways between high-risk and low-risk groups. NS, no significant; *p < 0.05; **p < 0.01; ***p < 0.001.

To clarify the factors affecting tumor immunogenicity between the two risk groups, we initially examined the neoantigen load, TCR diversity, mutation rate, BCR diversity, CNV burden, aneuploidy, and intertumoral heterogeneity. Compared to the high-risk group (all p < 0.05), the low-risk group harbored a higher mutation rate and neoantigen burden alongside significantly greater BCR and TCR diversity (all p < 0.05) (**Figure 6A**). However, the high-risk group exhibited significantly higher aneuploidy and CNV burdens (all p < 0.05) (**Figure 6A**). This aligns with existing research associating tumor aneuploidy with dampened immunotherapy responses (41). Compared to the low-risk group, individuals in the high-risk group exhibited significantly greater intertumoral heterogeneity (p < 0.05) (**Figure 6A**). This finding aligns with the hypothesis that tumors, facing a diminished immune response, may evolve clonally, leading to increased heterogeneity. This suggests that the heightened immunogenicity in the low-risk group might trigger an extrinsic immune response. To further explore the underlying mutational processes, we profiled mutational signatures based on somatic mutation data in both groups. This analysis revealed two distinct mutagenic patterns within the TCGA cohort (**Figure 6B**). The low-risk group exhibited a higher prevalence of SBS6, a mutational signature associated with defective DNA mismatch repair (**Figure 6C**). We further analyzed oncogene enrichment in ten key pathways, revealing distinct patterns. Whereas the cell cycle and Wnt pathways were enriched in the high-risk group (potentially linked to immune exclusion) (42), the Notch, PI3K, RAS, TGF beta, and TP53 pathways showed higher activity in the low-risk group (**Figure 6D**).

### Copy number features of the GMS

**Figure 7:**
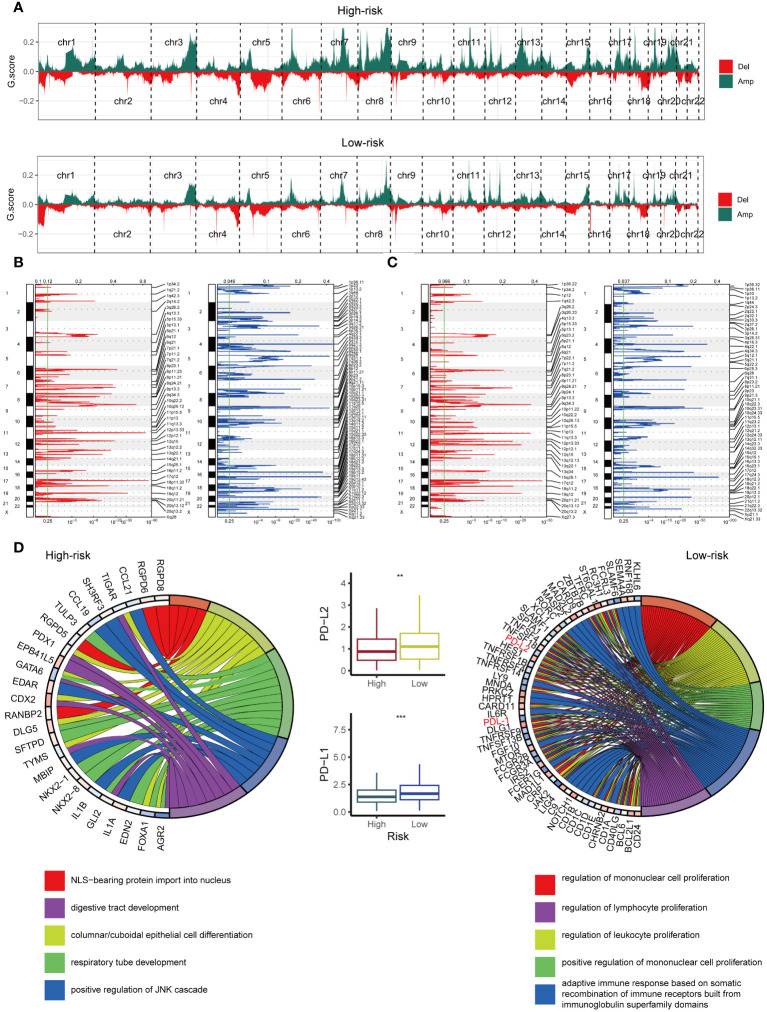
Examination of Copy Number Alterations in High-Risk and Low-Risk Groups. **(A)** Displaying copy number profiles for the high-risk group (upper) and low-risk group (lower). **(B)** Elaborating on cytobands with focal amplifications (left) and deletions (right) peaks identified within the high-risk group. **(C)** Exploring cytobands with focal amplifications (left) and deletions (right) peaks detected in the low-risk group. **(D)** Circular plot showcasing the top 5 biological processes along with their corresponding enriched genes in the high-risk (left) and low-risk (right) groups. Additionally, comparing the mRNA expression of PD-L1 and PD-L2 between the high-risk and low-risk cohorts from TCGA (middle). **p < 0.01; ***p < 0.001.

The high-risk and low-risk groups harbored vastly different chromosomal abnormalities (**Figure 7A**). Notably, the low-risk group, unlike the high-risk group (**Figures 7B, C**), exhibited focal amplifications of immune genes, including PD-L1 (9p24.1) and PD-L2 (9p24.1). While 625 amplified genes were shared between the groups, the high-risk and low-risk groups harbored 373 and 1597 unique amplified genes, respectively. We further analyzed these amplified genes using Gene Ontology (GO) biological processes (**Figure 7D**). The GO enrichment analysis revealed a different pattern in the low-risk group (**Figure 7D**), including five immune-related processes focused on cell proliferation (mononuclear, lymphocyte, and leukocyte) and adaptive immunity through immunoglobulin superfamily domain recombination. Notably, no such immune pathways enrichment was observed in the high-risk group (**Figure 7D**). Intriguingly, PD-L1 and PD-L2, key players in immune modulation, reside within the 9p24.1 amplification peak unique to the low-risk group, suggesting their potential contribution to the observed enhanced immune response. Consistent with this, mRNA expression of PD-L1 and PD-L2 mirrored the CNV pattern, with their levels being significantly higher in the low-risk group (**Figure 7D**), highlighting the influence of tumor copy number variations on immune infiltration patterns.

### Identification of small molecule drugs negatively associated with GMS

**Figure 8:**
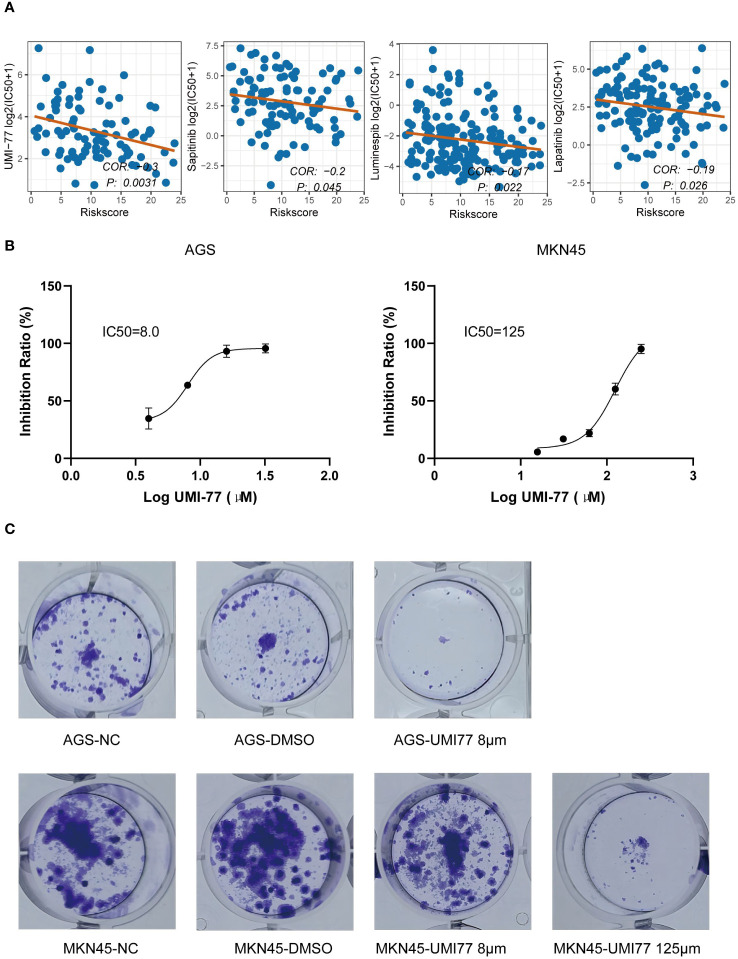
Identification of small molecule drugs negatively associated with GMS. **(A)** Correlation of half maximal inhibitory concentration (IC50) with GMS for UMI-77, Luminespib, Lapatinib, and Sapitinib. **(B)** IC50 of UMI-77 of AGS (right) and MKN45 (left). **(C)** Clonogenicity of AGS (above) and MKN45 (below) by using a colony-forming assay.

Based on the GDSC database, we identified that UMI-77, luminespib, lapatinib, and sapitinib exhibited the lowest p-values in the correlation test between GMS score and IC50, with UMI-77 having the smallest p-value (p < 0.05) (**Figure 8A**). We inferred that UMI-77 could be more effective for high-risk patients. To test this hypothesis, we measured the GMS of two cell lines in our laboratory (GMS score of AGS: 17.91; GMS score of MKN45: 4.43) and compared their sensitivity to UMI-77. The IC50 of UMI-77 for AGS and MKN45 was 8μM and 125μM, respectively (**Figure 8B**). A plate clone formation assay confirmed that AGS was more sensitive to UMI-77 (**Figure 8C**).

## Discussion

A genomic classifier named GMS, consisting of 23 genes, was developed and validated. It was derived from an artificial intelligence network aimed at enhancing the prediction of ICIs therapy efficacy in gastrointestinal cancer patients. The selection of the most efficient model involved utilizing a combination of VSOLassoBag and RSF methods, which displayed the highest average C-index in the test cohorts. The GMS had a prognostic value independent of other factors and showed consistent performance in the validation cohorts. ROC analysis also demonstrated that the GMS had high sensitivity and specificity in predicting 6/12/24 months OS and clinical response. The GMS exhibited a significantly superior level of predictive accuracy in comparison to both clinical attributes (e.g., sex) and molecular characteristics (e.g., MSI, TMB, and PD-L1 expression). This indicates the considerable potential for enhanced clinical translation and utilization of the GMS.

Leveraging the comprehensive data of the TCGA cohort, we delved into the diverse responses of cancers to immunotherapy treatment. The low-risk group stood out for its dense immune cell infiltration, rigorously supported by multiple algorithms. This internal immunological terrain was additionally fortified by potent immunogenic features: elevated mutation rates and a substantial neoantigen burden. In contrast to the high-risk group, the low-risk group also exhibited increased expression of immune checkpoint proteins such as PD-L1, PD-1, and CTLA-4, which could contribute to a more favorable response to ICIs therapy. The activated antitumor immunity, elevated PD-L1, PD-L2 and CTLA-4 expression, and heightened tumor immunogenicity likely explain why the low-risk group benefits from ICI therapy compared to their high-risk counterparts.

Our research offers the following novel contributions and practical implications. Firstly, we have developed an artificial intelligence network that comprises 297 algorithm combinations. This integration encompasses 22 algorithms, drawn from traditional regression, machine learning, and deep learning methodologies. This network featured a diverse and comprehensive set of algorithms, and exhibited superior predictive performance than previous studies (17, 25). Moreover, the optimal combination was VSOLassoBag and RSF, which was not considered in the prior study (25). The dimensionality of the variables was further reduced by the additional algorithm combinations, making the GMS more simplified and feasible. Secondly, the creation of multibiomarker predictive models demands a thorough comprehension of the elements impacting the dependability and precision of high-throughput assays in clinical scenarios. The variability in biomarker measurements, particularly those that is technical (platform-dependent), is a critical concern. A number of mRNA-based signatures have been developed to forecast clinical efficacy for patients receiving ICI therapy, including the T cell-inflamed gene-expression profile (GEP), which comprises an 18-gene panel (43). The evaluation of mRNA expression is carried out through relative quantification by normalizing it to reference genes (44). The risk scoring and threshold values of mRNA signatures may not be directly applicable for validation with diverse measurement data types. In this study, we have identified specific gene mutations to forecast the clinical effectiveness of ICIs. Consequently, the GMS is resilient to technical variations, even when different platforms are employed across various centers. Thirdly, in clinical practice, the GMS aids in avoiding potential immune-related adverse effects for patients who are unlikely to respond, and it enables the early identification of patients who may benefit from more effective therapies. Additionally, given that the average cost of a treatment regimen often exceeds $120,000 (45), implementing biomarker strategies that improve diagnostic precision can help prevent significant costs for treatments with limited expected benefits. In summary, since obtaining tumor specimens through targeted next-generation sequencing (NGS) of these genes is simpler and less costly compared to assessing TMB, which are complex and expensive in routine practice, the GMS with these refinement merits evaluation. Such an assessment could enhance diagnostic accuracy and cost-effectiveness in clinic.

Utilizing the GDSC, we identified UMI-77, a small molecule drug that demonstrated the most significant p-value and a strong negative correlation with GMS. UMI-77 is an FDA-approved candidate drug for pancreatic cancer, known to inhibit cell proliferation and induce apoptosis in pancreatic cancer cells (46). Moreover, UMI-77 triggers mitophagy, a process that selectively eliminates damaged mitochondria, making it a potential therapeutic option for Alzheimer’s disease (47, 48) and glioma (49). Our observations revealed that the AGS cell line, categorized in the high-risk group, displayed greater sensitivity to UMI-77 than the MKN45 cell line, which belongs to the low-risk group. Based on these findings, we hypothesize that combining UMI-77 with ICIs may enhance the efficacy of ICIs in the high-risk group. However, this hypothesis necessitates further validation through *in vivo* experiments.

### Limitations

Our research is not without limitations, which are important to acknowledge. Firstly, we did not have access to comprehensive clinical records for all patients, potentially introducing bias in the data analysis. Secondly, the inclusion of diverse gastrointestinal cancer types and the retrospective nature of the study may have introduced confounding factors. Thirdly, the abundance of immune cells and the expression of immune checkpoints should be substantiated through immunohistochemistry techniques. To address these limitations, further analysis and validation are needed through prospective studies involving a large cohort of gastrointestinal cancer patients with diverse ethnic backgrounds receiving ICIs therapy. Such studies would help strengthen the findings and implications of our research.

## Conclusions

In summary, our GMS emerges as a promising biomarker for both prognosis and prediction of ICI treatment response in gastrointestinal cancer patients. This signature also presents an economical approach to pinpoint patients who may benefit from immunotherapy, a concept that should be further explored through prospective research. The GMS could significantly contribute to the refinement of personalized treatment plans and the enhancement of patient outcomes in gastrointestinal cancer immunotherapy.

## Data availability statement

The genomic and clinical data for the MSK cohort, Janjigian and Pender cohort, and PUCH cohort are openly available and were downloaded from the following sources: MSK cohort: http://www.cbioportal.org/study?id=tmb_mskcc_2018; Janjigian cohort: https://www.cbioportal.org/study/summary?id=egc_msk_2017; Pender cohort: http://clincancerres.aacrjournals.org/content/27/1/202.article-info; PUCH cohort: https://www.bcgsc.ca/downloads/immunoPOG/; The DNA-seq and RNA-seq data from the TCGA dataset is available for download at https://portal.gdc.cancer.gov/.

## Ethics statement

Ethical approval was not required for the studies on humans in accordance with the local legislation and institutional requirements because only commercially available established cell lines were used.

## Author contributions

BY: Methodology, Software, Visualization, Writing – original draft, Writing – review & editing. ZL: Methodology, Software, Visualization, Writing – original draft, Writing – review & editing. QW: Conceptualization, Data curation, Methodology, Software, Writing – original draft, Writing – review & editing.

## References

[1] Arnold M Abnet CC Neale RE Vignat J Giovannucci EL McGlynn KA. Global burden of 5 major types of gastrointestinal cancer. Gastroenterology. (2020) 159:335–349.e315. doi: 10.1053/j.gastro.2020.02.068 32247694 PMC8630546

[2] Carlino MS Larkin J Long GV. Immune checkpoint inhibitors in melanoma. Lancet (London England). (2021) 398:1002–14. doi: 10.1016/S0140-6736(21)01206-X 34509219

[3] Lu Z Peng Z Liu C Wang Z Wang Y Jiao X. Current status and future perspective of immunotherapy in gastrointestinal cancers. Innovation (Cambridge (Mass)). (2020) 1:100041. doi: 10.1016/j.xinn.2020.100041 34557714 PMC8454608

[4] Rao D Parakrama R Augustine T Liu Q Goel S Maitra R. Immunotherapeutic advances in gastrointestinal Malignancies. NPJ Precis Oncol. (2019) 3:4. doi: 10.1038/s41698-018-0076-8 30729176 PMC6363766

[5] Dudley JC Lin MT Le DT Eshleman JR. Microsatellite instability as a biomarker for PD-1 blockade. Clin Cancer Res. (2016) 22:813–20. doi: 10.1158/1078-0432.CCR-15-1678 26880610

[6] Asaoka Y Ijichi H Koike K. PD-1 blockade in tumors with mismatch-repair deficiency. New Engl J Med. (2015) 373:1979. doi: 10.1056/NEJMc1510353 26559583

[7] Fuchs CS Doi T Jang RW Muro K Satoh T MaChado M. Safety and efficacy of pembrolizumab monotherapy in patients with previously treated advanced gastric and gastroesophageal junction cancer: phase 2 clinical KEYNOTE-059 trial. JAMA Oncol. (2018) 4:e180013. doi: 10.1001/jamaoncol.2018.0013 29543932 PMC5885175

[8] Wang F Wei XL Wang FH Xu N Shen L Dai GH. Safety, efficacy and tumor mutational burden as a biomarker of overall survival benefit in chemo-refractory gastric cancer treated with toripalimab, a PD-1 antibody in phase Ib/II clinical trial NCT02915432. Ann Oncol. (2019) 30:1479–86. doi: 10.1093/annonc/mdz197 PMC6771223 31236579

[9] Huang J Xu J Chen Y Zhuang W Zhang Y Chen Z. Camrelizumab versus investigator's choice of chemotherapy as second-line therapy for advanced or metastatic oesophageal squamous cell carcinoma (ESCORT): a multicentre, randomised, open-label, phase 3 study. Lancet Oncol. (2020) 21:832–42. doi: 10.1016/S1470-2045(20)30110-8 32416073

[10] Marabelle A Le DT Ascierto PA Di Giacomo AM De Jesus-Acosta A Delord JP. Efficacy of pembrolizumab in patients with noncolorectal high microsatellite instability/Mismatch repair-Deficient cancer: results from the phase II KEYNOTE-158 study. J Clin Oncol. (2020) 38:1–10. doi: 10.1200/JCO.19.02105 31682550 PMC8184060

[11] Samstein RM Lee CH Shoushtari AN Hellmann MD Shen R Janjigian YY. Tumor mutational load predicts survival after immunotherapy across multiple cancer types. Nat Genet. (2019) 51:202–6. doi: 10.1038/s41588-018-0312-8 PMC6365097 30643254

[12] Xue L Tang W Zhou J Xue J Li Q Ge X. Next-generation sequencing identifies CDKN2A alterations as prognostic biomarkers in recurrent or metastatic head and neck squamous cell carcinoma predominantly receiving immune checkpoint inhibitors. Front Oncol. (2023) 13:1276009. doi: 10.3389/fonc.2023.1276009 37936609 PMC10627168

[13] Zhu G Shi R Li Y Zhang Z Xu S Chen C. ARID1A, ARID1B, and ARID2 mutations serve as potential biomarkers for immune checkpoint blockade in patients with non-small cell lung cancer. Front Immunol. (2021) 12:670040. doi: 10.3389/fimmu.2021.670040 34512623 PMC8426508

[14] Hu X Xu H Xue Q Wen R Jiao W Tian K. The role of ERBB4 mutations in the prognosis of advanced non-small cell lung cancer treated with immune checkpoint inhibitors. Mol Med (Cambridge Mass). (2021) 27:126. doi: 10.1186/s10020-021-00387-z 34620079 PMC8496027

[15] Zhang J Zhou N Lin A Luo P Chen X Deng H. ZFHX3 mutation as a protective biomarker for immune checkpoint blockade in non-small cell lung cancer. Cancer immunology immunotherapy: CII. (2021) 70:137–51. doi: 10.1007/s00262-020-02668-8 PMC10992006 32653938

[16] Wang Z Zhang Q Qi C Bai Y Zhao F Chen H. Combination of AKT1 and CDH1 mutations predicts primary resistance to immunotherapy in dMMR/MSI-H gastrointestinal cancer. J immunotherapy Cancer. (2022) 10:e004703. doi: 10.1136/jitc-2022-004703 PMC9204428 35705314

[17] Jiao X Wei X Li S Liu C Chen H Gong J. A genomic mutation signature predicts the clinical outcomes of immunotherapy and characterizes immunophenotypes in gastrointestinal cancer. NPJ Precis Oncol. (2021) 5:36. doi: 10.1038/s41698-021-00172-5 33947957 PMC8096820

[18] Shim JH Kim HS Cha H Kim S Kim TM Anagnostou V. HLA-corrected tumor mutation burden and homologous recombination deficiency for the prediction of response to PD-(L)1 blockade in advanced non-small-cell lung cancer patients. Ann Oncol. (2020) 31:902–11. doi: 10.1016/j.annonc.2020.04.004 32320754

[19] Wang Z Ge Y Li H Fei G Wang S Wei P. Identification and validation of a genomic mutation signature as a predictor for immunotherapy in NSCLC. Bioscience Rep. (2022) 42. doi: 10.1042/BSR20220892 PMC9702799 36305643

[20] Bai X Wu DH Ma SC Wang J Tang XR Kang S. Development and validation of a genomic mutation signature to predict response to PD-1 inhibitors in non-squamous NSCLC: a multicohort study. J immunotherapy Cancer. (2020) 8:e000381. doi: 10.1136/jitc-2019-000381 PMC7328897 32606052

[21] Qiu T Shi X Wang J Li Y Qu S Cheng Q. Deep learning: A rapid and efficient route to automatic metasurface design. Advanced Sci (Weinheim Baden-Wurttemberg Germany). (2019) 6:1900128. doi: 10.1002/advs.201900128 PMC6662056 31380164

[22] Holder LB Haque MM Skinner MK. Machine learning for epigenetics and future medical applications. Epigenetics. (2017) 12:505–14. doi: 10.1080/15592294.2017.1329068 PMC5687335 28524769

[23] Zhao S Wang L Ding W Ye B Cheng C Shao J. Crosstalk of disulfidptosis-related subtypes, establishment of a prognostic signature and immune infiltration characteristics in bladder cancer based on a machine learning survival framework. Front Endocrinol. (2023) 14:1180404. doi: 10.3389/fendo.2023.1180404 PMC10154596 37152941

[24] Ye B Wang Q Zhu X Zeng L Luo H Xiong Y. Single-cell RNA sequencing identifies a novel proliferation cell type affecting clinical outcome of pancreatic ductal adenocarcinoma. Front Oncol. (2023) 13:1236435. doi: 10.3389/fonc.2023.1236435 37601684 PMC10433893

[25] Liu Z Liu L Weng S Guo C Dang Q Xu H. Machine learning-based integration develops an immune-derived lncRNA signature for improving outcomes in colorectal cancer. Nat Commun. (2022) 13:816. doi: 10.1038/s41467-022-28421-6 35145098 PMC8831564

[26] Janjigian YY Sanchez-Vega F Jonsson P Chatila WK Hechtman JF Ku GY. Genetic predictors of response to systemic therapy in esophagogastric cancer. Cancer Discovery. (2018) 8:49–58. doi: 10.1158/2159-8290.CD-17-0787 29122777 PMC5813492

[27] Pender A Titmuss E Pleasance ED Fan KY Pearson H Brown SD. Genome and transcriptome biomarkers of response to immune checkpoint inhibitors in advanced solid tumors. Clin Cancer Res. (2021) 27:202–12. doi: 10.1158/1078-0432.CCR-20-1163 33020056

[28] Rizvi NA Hellmann MD Snyder A Kvistborg P Makarov V Havel JJ. Cancer immunology. Mutational landscape determines sensitivity to PD-1 blockade in non-small cell lung cancer. Sci (New York NY). (2015) 348:124–8. doi: 10.1126/science.aaa1348 PMC4993154 25765070

[29] Thorsson V Gibbs DL Brown SD Wolf D Bortone DS Ou Yang TH. The immune landscape of cancer. Immunity. (2018) 48:812–30.e814. doi: 10.1016/j.immuni.2018.03.023 29628290 PMC5982584

[30] Finotello F Mayer C Plattner C Laschober G Rieder D Hackl H. Molecular and pharmacological modulators of the tumor immune contexture revealed by deconvolution of RNA-seq data. Genome Med. (2019) 11:34. doi: 10.1186/s13073-019-0638-6 31126321 PMC6534875

[31] Racle J Gfeller D. EPIC: A tool to estimate the proportions of different cell types from bulk gene expression data. Methods Mol Biol (Clifton NJ). (2020) 2120:233–48. doi: 10.1007/978-1-0716-0327-7_17 32124324

[32] Becht E Giraldo NA Lacroix L Buttard B Elarouci N Petitprez F. Erratum to: Estimating the population abundance of tissue-infiltrating immune and stromal cell populations using gene expression. Genome Biol. (2016) 17:249. doi: 10.1186/s13059-016-1113-y 27908289 PMC5134277

[33] Yoshihara K Shahmoradgoli M Martínez E Vegesna R Kim H Torres-Garcia W. Inferring tumour purity and stromal and immune cell admixture from expression data. Nat Commun. (2013) 4:2612. doi: 10.1038/ncomms3612 24113773 PMC3826632

[34] He Y Jiang Z Chen C Wang X. Classification of triple-negative breast cancers based on Immunogenomic profiling. J Exp Clin Cancer research: CR. (2018) 37:327. doi: 10.1186/s13046-018-1002-1 30594216 PMC6310928

[35] Rooney MS Shukla SA Wu CJ Getz G Hacohen N. Molecular and genetic properties of tumors associated with local immune cytolytic activity. Cell. (2015) 160:48–61. doi: 10.1016/j.cell.2014.12.033 25594174 PMC4856474

[36] Hänzelmann S Castelo R Guinney J. GSVA: gene set variation analysis for microarray and RNA-seq data. BMC Bioinf. (2013) 14:7. doi: 10.1186/1471-2105-14-7 PMC3618321 23323831

[37] Yu G Wang LG Han Y He QY. clusterProfiler: an R package for comparing biological themes among gene clusters. Omics: J Integr Biol. (2012) 16:284–7. doi: 10.1089/omi.2011.0118 PMC3339379 22455463

[38] Zhou Y Zhou B Pache L Chang M Khodabakhshi AH Tanaseichuk O. Metascape provides a biologist-oriented resource for the analysis of systems-level datasets. Nat Commun. (2019) 10:1523. doi: 10.1038/s41467-019-09234-6 30944313 PMC6447622

[39] Sanchez-Vega F Mina M Armenia J Chatila WK Luna A La KC. Oncogenic signaling pathways in the cancer genome atlas. Cell. (2018) 173:321–37.e310. doi: 10.1016/j.cell.2018.03.035 29625050 PMC6070353

[40] Wu W Liu Y Zeng S Han Y Shen H. Intratumor heterogeneity: the hidden barrier to immunotherapy against MSI tumors from the perspective of IFN-γ signaling and tumor-infiltrating lymphocytes. J Hematol Oncol. (2021) 14:160. doi: 10.1186/s13045-021-01166-3 34620200 PMC8499512

[41] Davoli T Uno H Wooten EC Elledge SJ. Tumor aneuploidy correlates with markers of immune evasion and with reduced response to immunotherapy. Sci (New York NY). (2017) 355:eaaf8399. doi: 10.1126/science.aaf8399 PMC5592794 28104840

[42] Luke JJ Bao R Sweis RF Spranger S Gajewski TF. WNT/β-catenin pathway activation correlates with immune exclusion across human cancers. Clin Cancer Res. (2019) 25:3074–83. doi: 10.1158/1078-0432.CCR-18-1942 PMC6522301 30635339

[43] Ott PA Bang YJ Piha-Paul SA Razak ARA Bennouna J Soria JC. T-cell-inflamed gene-expression profile, programmed death ligand 1 expression, and tumor mutational burden predict efficacy in patients treated with pembrolizumab across 20 cancers: KEYNOTE-028. J Clin Oncol. (2019) 37:318–27. doi: 10.1200/JCO.2018.78.2276 30557521

[44] Long J Wang D Wang A Chen P Lin Y Bian J. A mutation-based gene set predicts survival benefit after immunotherapy across multiple cancers and reveals the immune response landscape. Genome Med. (2022) 14:20. doi: 10.1186/s13073-022-01024-y 35197093 PMC8867854

[45] Lu S Stein JE Rimm DL Wang DW Bell JM Johnson DB. Comparison of biomarker modalities for predicting response to PD-1/PD-L1 checkpoint blockade: A systematic review and meta-analysis. JAMA Oncol. (2019) 5:1195–204. doi: 10.1001/jamaoncol.2019.1549 PMC6646995 31318407

[46] Abulwerdi F Liao C Liu M Azmi AS Aboukameel A Mady AS. A novel small-molecule inhibitor of mcl-1 blocks pancreatic cancer growth *in vitro* and *in vivo* . Mol Cancer Ther. (2014) 13:565–75. doi: 10.1158/1535-7163.MCT-12-0767 PMC4174574 24019208

[47] Cen X Chen Y Xu X Wu R He F Zhao Q. Pharmacological targeting of MCL-1 promotes mitophagy and improves disease pathologies in an Alzheimer's disease mouse model. Nat Commun. (2020) 11:5731. doi: 10.1038/s41467-020-19547-6 33184293 PMC7665171

[48] Cen X Xu X Xia H. Targeting MCL1 to induce mitophagy is a potential therapeutic strategy for Alzheimer disease. Autophagy. (2021) 17:818–9. doi: 10.1080/15548627.2020.1860542 PMC8032245 33342330

[49] Chen C Zhu S Zhang X Zhou T Gu J Xu Y. Targeting the synthetic vulnerability of PTEN-Deficient glioblastoma cells with MCL1 inhibitors. Mol Cancer Ther. (2020) 19:2001–11. doi: 10.1158/1535-7163.MCT-20-0099 32737157

