# Supplementary material for: A novel artificial intelligence network to assess the prognosis of gastrointestinal cancer to immunotherapy based on genetic mutation features
Source: Front Immunol. 2024 Jun 27;15:1428529. doi: 10.3389/fimmu.2024.1428529 (PMC11236566; doi:10.3389/fimmu.2024.1428529)
Supplement: Supplementary file 1 [file Table_1.docx]

|  | **MSK** | **Janjigian&Pender**  **en**der **cohort** | **PUCH** |
| --- | --- | --- | --- |
| **No. of patients** | 233 | 48 | 91 |
| **Age (median, range)** | 59(19-87) | 59(22-86) | 59(15-76) |
| **Gender (%)** |  |  |  |
| Female | 74(31.8%) | 11(22.9%) | 25(27.5%) |
| male | 159(68.4%) | 37(77.1%) | 66(72.5%) |
| **Drug Type(%)** |  |  |  |
| Combo | 41(17.6%) | NA | 11(12.1%) |
| Single | 192(82.4%) | NA | 80(87.9%) |
| unKown | 0(0%) | 48(100%) | 0(0%) |
| **TMB (%)** |  |  |  |
| ≥10 | 62(26.6%) | 13(27.1%) | 21(23.1%) |
| ＜10 | 171(73.4%) | 35(72.9%) | 70(76.9%) |
| **PD-L1 (%)** |  |  |  |
| Neg | NA | 6(12.5%) | 28(30.77%) |
| Pos | NA | 13(27.1%) | 33(36.3%) |
| unKown | 233(100%) | 29(60.4%) | 30(32.97%) |
| **MSI (%)** |  |  |  |
| MSS | NA | 34(70.8%) | 48(52.7%) |
| MSI-H | NA | 5(10.4%) | 22(24.2%) |
| unKown | 233(100%) | 9(9.9%) | 21(23.1%) |

**Supplementary Table 1.** The clinical information of the three cohorts.
